# Estimating the epidemic consequences of HIV prevention gaps among key populations

**DOI:** 10.1002/jia2.25739

**Published:** 2021-06-30

**Authors:** Sharmistha Mishra, Romain Silhol, Jesse Knight, Refilwe Phaswana‐Mafuya, Daouda Diouf, Linwei Wang, Sheree Schwartz, Marie‐Claude Boily, Stefan Baral

**Affiliations:** ^1^ Department of Medicine University of Toronto Toronto ON Canada; ^2^ Institute of Medical Sciences University of Toronto Toronto ON Canada; ^3^ Institute of Health Policy, Management and Evaluation University of Toronto Toronto On Canada; ^4^ Li Ka Shing Knowledge Institute St. Michael’s Hospital Unity Health Toronto Toronto ON Canada; ^5^ MRC Centre for Global Infectious Disease Analysis School of Public Health Imperial College London London United Kingdom; ^6^ University of Johannesburg Johannesburg Gauteng South Africa; ^7^ Enda Santé Dakar Senegal; ^8^ Department of Epidemiology Johns Hopkins School of Public Health Baltimore MD USA

**Keywords:** Mathematical model, HIV/AIDS, HIV transmission, population attributable fraction, key populations

## Abstract

**Introduction:**

HIV epidemic appraisals are used to characterize heterogeneity and inequities in the context of the HIV pandemic and the response. However, classic measures used in appraisals have been shown to underestimate disproportionate risks of onward transmission, particularly among key populations. In response, a growing number of modelling studies have quantified the consequences of unmet prevention and treatment needs (prevention gaps) among key populations as a transmission population attributable fraction over time (tPAF_t_). To aid its interpretation and use by programme implementers and policy makers, we outline and discuss a conceptual framework for understanding and estimating the tPAF_t_ via transmission modelling as a measure of onward transmission risk from HIV prevention gaps; and discuss properties of the tPAF_t_.

**Discussion:**

The distribution of onward transmission risks may be defined by who is at disproportionate risk of onward transmission, and under which conditions. The latter reflects prevention gaps, including secondary prevention via treatment: the epidemic consequences of which may be quantified by the tPAF_t_. Steps to estimating the tPAF_t_ include parameterizing the acquisition and onward transmission risks experienced by the subgroup of interest, defining the most relevant counterfactual scenario, and articulating the time‐horizon of analyses and population among whom to estimate the relative difference in cumulative transmissions; such steps could reflect programme‐relevant questions about onward transmission risks. Key properties of the tPAF_t_ include larger onward transmission risks over longer time‐horizons; seemingly mutually exclusive tPAF_t_ measures summing to greater than 100%; an opportunity to quantify the magnitude of disproportionate onward transmission risks with a per‐capita tPAF_t_; and that estimates are conditional on what has been achieved so far in reducing prevention gaps and maintaining those conditions moving forward as the status quo.

**Conclusions:**

The next generation of HIV epidemic appraisals has the potential to support a more specific HIV response by characterizing heterogeneity in disproportionate risks of onward transmission which are defined and conditioned on the past, current and future prevention gaps across subsets of the population.

## 1 INTRODUCTION

HIV epidemic appraisals comprise frameworks to characterize a local epidemic in order to help shape the response [1, 2, 3]. For example, appraisals have been used to help guide prioritization of subgroups for tailored interventions via the strategic design of programmes and services [1, 4]. Over the past four decades, there has been an evolution in appraisals from classifications of epidemic type [5] and estimates of who acquires new infections [6], to transmission modelling specifically designed to characterize local transmission dynamics (or sources of transmission) and more explicitly consider underserved populations [7, 8, 9, 10, 11, 12, 13, 14, 15]. This evolution coincided with a growing call to increase the specificity of the HIV response, moving from largely uniform strategies to prioritization with tailored strategies to address unmet HIV prevention and treatment needs (prevention gaps) across subgroups [16, 17, 18, 19, 20, 21].

Mathematical models have provided important insights and estimates of the impact and efficiency of HIV interventions prioritized or tailored to key populations, such as individuals engaged in sex work, men who have sex with men and persons who inject drugs [15, 22, 23, 24, 25]. At the same time, transmission models have also been specifically designed and used for local HIV epidemic appraisals, an objective that is distinct yet complementary to modelling the impact or efficiency of interventions. For example, between 2010 and 2020, at least eight published studies examined and explicitly reported estimates of onward transmission stemming from the prevention gaps across subgroups in sub‐Saharan Africa [7, 8, 9, 10, 11, 12, 13, 14]. The studies simulated the causal pathway from the unmet needs among a relatively few to population‐level transmission via chains of transmission over sexual networks. The studies found that 19% to 40% and 4% to 64% of onward transmission over a ten‐year time‐horizon, after at least 2005, stem from unmet HIV prevention and treatment needs among female sex workers (FSW) and men who have sex with men respectively [7, 8, 9, 10, 11, 12, 13, 14]. These estimates of onward transmission risks have been referred to as the transmission population attributable fraction over time (tPAF_t_), which quantify the epidemic consequences of the prevention gaps among subgroups.

The tPAF_t_ extends on the established construct of the population attributable fraction as first described – i.e. what would happen in the absence of a given risk factor (i.e. what is the counterfactual?) [26]. Under this established construct, the tPAF_t_ explicitly captures chains of transmission (e.g. transmissions to partners’ partners, and so on), which cannot be captured with purely observational data and inputs such as prevalence ratios commonly used to estimate population attributable fractions [26, 27]. Similarly, the distribution of annual new infections acquired by FSW or distribution of annual transmissions among FSW have been shown to underestimate the tPAF_t_ of prevention gaps among FSW in the medium to long‐term [13, 28, 29, 30]. That is, short‐term metrics of infection have been shown to underestimate the downstream consequences of the prevention gaps among key populations. Furthermore, the modelling studies found that the potential transmission impact of allocating finite resources on the basis of disproportionate risks of onward transmission could be greater than allocation guided by the distribution of who acquired infection [13, 14, 29, 30].

A summary of minimum data needs for estimating the tPAF_t_ has been previously described [31]. However, there are challenges in interpretation. For example, estimates among key populations may be smaller than expected [10] and different studies may use different counterfactuals when estimating the tPAF_t_ for their particular study. Thus, we outline a conceptual framework to help support the design and interpretation of tPAF_t_ estimates from transmission models for programme implementers and policy makers looking to increase the specificity of their local HIV response.

## 2 DISCUSSION

The proposed conceptual framework for the design and interpretation of tPAF_t_ estimates could begin with three problem/objective statements [32]: (1) who is at disproportionate risk of onward transmission; (2) under what prevention gaps and (3) among whom are onward transmission counted and over what time period. For example, we could estimate a tPAF_t_ to measure the contribution of unmet prevention needs (question 2) among FSW (question 1) to all new HIV infections of the total population over the next ten years (question 3). We discuss the details of the three questions with examples of tPAF_t_ estimates using published modelling results, followed by the implications of the results. We then summarize a list of key properties of the tPAF_t_.

**Figure 1:**
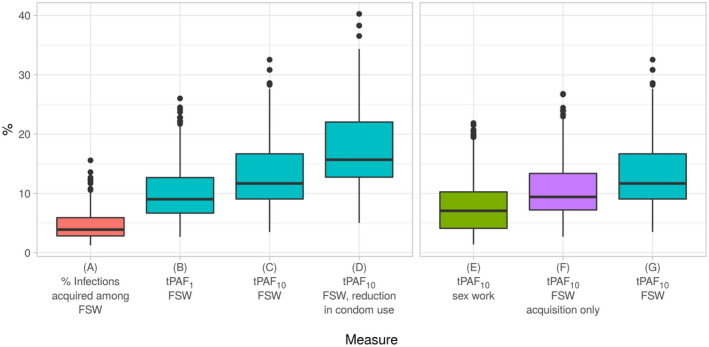
Distribution of various estimates of the tPAF_t_ related to sex work in Yaoundé, Cameroon [13]. The Yaoundé transmission model included FSW, clients of FSW, men who have sex with men and lower activity males and females. The model was used to generate the measures referred to in the main text. **(A)** is the percent of new infections acquired by FSW in the status quo scenario (not a tPAF_t_ measure). **(B** and **C)** are the cumulative percentage of infections that stem from prevention gaps experienced by FSW, and estimated by interrupting acquisition and transmission across all partnerships among FSW from 2019 to 2020 (B, tPAF_1_) and from 2019 to 2029 (**C**, tPAF_10_). **(D)** is the same measure as C except that condom‐use among FSW declines after 2019 such that the conditions under which the tPAF_10_ was estimated in (**C)**, no longer holds. Examples of different counterfactuals are shown with E‐G for the tPAF_10_: In E, the transmission was set to zero (“turned off”) in the context of sex work alone. In F, only acquisition risks among FSW were set to zero, whereas in **(G)** the acquisition and transmission across all partnerships of FSW were set to zero. The tPAF_10_ of sex work **(E)** was smaller than the tPAF_10_ of acquisition risks among FSW **(F)**, because in 2019, levels of condom use between FSW and their non‐paying partners were lower than condom use in the context of sex work [13]. tPAF_t_ (transmission population attributable fraction); FSW (female sex workers).

**Figure 2:**
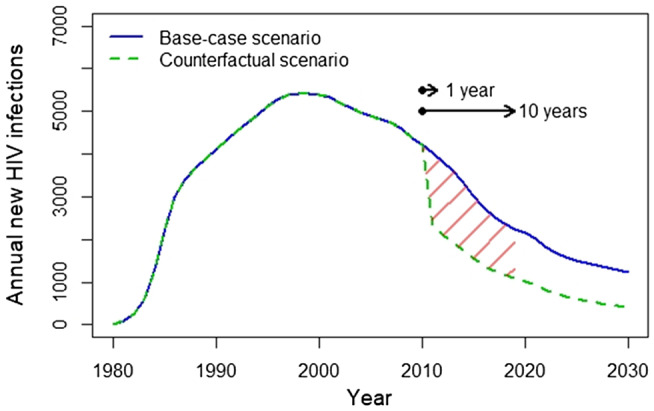
Illustration of base‐case and counterfactual scenarios used to estimate the transmission population attributable fraction (tPAF_t_) over one and ten years [13], based on an HIV model of Yaoundé, Cameroon. The counterfactual scenario represents “turning off” or interrupting transmission among all partnerships among FSW in the year 2010, and estimating the relative difference in cumulative infections in the total population up to 2011 and 2020 to generate the tPAF_1_ and tPAF_10_, respectively, of the prevention gaps among FSW. FSW (female sex workers).

We have chosen published analyses from Yaoundé, Cameroon, where trends in the prevalence of HIV amongst FSW and data on sexual partnerships and coverage of interventions are available to illustrate approaches [13]. The tPAF_t_ in relation sex work was modelled using cumulative HIV incidence over different periods. In Figure 1A‐D, the proportion of HIV infections acquired by FSW are compared with the proportion of infections prevented across the population if acquisition and transmission risks among FSW could be halted for one and ten years assuming high levels of condom use in the counterfactual, and then for ten years assuming condom use would otherwise decline. For example in Yaoundé, although FSW acquired 4% of infections in 2019, an estimated 12% (tPAF_1_) and 16% (tPAF_10_) of transmissions in the total population over the next one and ten years, respectively, are predicted to stem from the prevention gaps among FSW [13]. Figure 1E‐F depicts the results of using different counterfactuals when estimating the tPAF_t_ in relation to sex work, and thus, the different implications for interventions focused on particular risks in the context of sex work. Figure 2 illustrates the course of the HIV epidemic in the analysis for Yaoundé with and without halting acquisition and transmission risks among FSW.

### 2.1 Question 1. Who is at disproportionate risk of onward transmission? Relative acquisition and relative transmission risks

The factors that define disproportionate risk for a specific population may include those related to differential exposures and transmission networks, with intersections across these factors. Differential exposures and networks may be operationalized in different ways across models [33]. Relative risks may stem from variability in the number and types of exposures (e.g. types of partnerships and types sex acts by partnership type, in the context of sexual transmission), modes of exposures (e.g. sexual, shared use of needles for injecting drugs, etc.) and sexual or injecting network characteristics (e.g. “who has sex with whom”), duration of exposures (e.g. duration in sex work) and the relative population size of subgroups. Critical local data inputs include subgroup population size, partnership or injecting network data and trends in HIV prevalence and prevalence ratios across subgroups [31].

### 2.2 Question 2. What are the prevention gaps, and which counterfactuals were used?

On their own, differential exposures and networks lead to disproportionate onward transmission risks. Interventions are meant to reduce these disproportionate risks and indeed, with adequate levels of interventions, the tPAF_t_ of key populations may be small. Thus, it is the combination of the underlying differential exposures and networks alongside intervention access and uptake that leads to the concept of unmet prevention and treatment needs, or prevention gaps. Prevention gaps reflect the conditions under which onward transmission risks persist, for example as a result of lower coverage of HIV treatment and viral suppression across key populations as compared with the wider population [34]. Of note, the level of intervention coverage needed to minimize onward transmission risks will be different across subgroups, exposures or networks. That is there is a different epidemic consequence of the same proportion not virally suppressed among men who have sex with men, FSW, client of FSW and the wider populations [10, 13, 30].

Thus, the tPAF_t_ can be used to estimate the onward transmission stemming from a single‐specific prevention gap (e.g. partnership type, such as sex work partnerships), a combination of prevention gaps, or all prevention gaps associated with a specific subgroup. Calculating the tPAF_t_ then involves comparing the projected number of infections under two scenarios: a base‐case scenario reflecting status quo, and a counterfactual scenario reflecting the prevention gap under study. The counterfactual can either be modelled as setting a component of the force of infection to zero (turning off transmission completely) or setting a relative rate of a component of the force of infection to one (equalizing relative rates).

Figure 1B illustrates the tPAF_10_ of various prevention gaps in the context of sex work and FSW from previously published analyses from Yaoundé, Cameroon, wherein transmission was interrupted completely in the context of: sex work partnerships only (Figure 1E), acquisition risks among FSW only (Figure 1F), all partnerships types among FSW (often referred to as the tPAF_t_ of the acquisition and transmission risks of FSW or the prevention gaps among FSW, Figure 1G). Of note, the tPAF_10_ of sex work was smaller than the tPAF_10_ of acquisition risks among FSW, because in 2019, levels of condom use between FSW and their non‐paying partners were lower than condom use in the context of sex work [13]. The choice of specific counterfactual is guided by the question of interest, and how the tPAF_t_ estimates could be used to inform exposure‐specific or subgroup‐specific interventions.

### 2.3 Question 3. Among whom are onward transmissions counted, and over what time period?

Interpreting the tPAF_t_ involves examining when the counterfactual scenario diverged from the base‐case scenario (Figure 2), the time‐horizon for counting infections, and among whom infections are counted. That is the tPAF_t_ reflects a measure from one time point to another, by counting the cumulative difference in new infections between the base‐case scenario and the counterfactual, in the total population or a specific subgroup. For example, the tPAF_t_ of prevention gaps among FSW could be measured as the proportion of infections in the total population over the next year (Figure 1B), or over the next 10 years (Figure 1C). Thus, the tPAF_t_ may be estimated across any time‐horizon and among any combination of subgroups included in the transmission model, depending on the problem/objective statements in relation to the distribution of onward transmission risks.

### 2.4 Summary of key properties of the tPAF_t_

Box 1 lists the properties of measures of onward transmission risks which have been demonstrated across various studies [7, 8, 9, 10, 11, 12, 13, 14, 29, 30]. Because of how infections are passed on within chains of transmission, the distribution of acquisition (who acquires most of the new infections in a given time period) is not equal to the distribution of onward transmission [28] (Figure 1A). Similarly, when prevention gaps lead to more than one secondary infection on average, the tPAF_t_ increases over longer time‐horizons (Figure 1A) [8, 13]. That is the finding across studies that the tPAF_t_ of prevention gaps among FSW increase over time means that the epidemiologic consequences are larger over longer time‐horizons [8]. The tPAF_t_ of multiple prevention gaps can also sum to more than 100%, in contrast to the proportion of total infections acquired by each sub‐population. This is because downstream transmission can be interrupted through multiple pathways, similar to discussions surrounding the population attributable fraction in other health contexts wherein disease can be prevented in multiple ways [35]. That is, the same transmission chain could be interrupted by meeting the treatment and/or prevention needs of several subgroups, resulting in “double‐counting” of the prevented infections in the tPAFt of each subgroup and thus the potential to sum to more than 100%.

Box 1 **Properties of the transmission population attributable fraction over time.**

The distribution of acquisition across subgroups is different from the distribution of onward transmissions.

Distribution of onward transmission risks will vary by time‐horizon of analyses (i.e. time‐period over which the consequences of unmet needs are estimated)

Distribution of onward transmission risks from past, current and future prevention gaps will be different because the epidemic conditions under which the tPAF_t_ is estimated may be different.

Distribution of onward transmission risks is conditional on what has been achieved so far and maintaining those conditions over the time‐horizon of analyses.

Summing the tPAF_t_ of multiple unmet treatment or prevention needs can be greater than 100% because multiple interventions can interrupt the same transmission chain and thereby prevent the same downstream infections.

Per‐capita estimates of tPAF_t_ as a measure of disproportionate onward transmission risk can help inform efficiencies in addressing prevention gaps.

tPAF_t,_ transmission population attributable fraction over time t.

The tPAF_t_ varies by context, including calendar time, because the conditions (prevention gaps) may have been different at different calendar times due to what had been achieved so far and the natural dynamics of an epidemic. That is the tPAF_t_ moving forward is conditional on what has been achieved so far and then maintaining those conditions (e.g. interventions) moving forward. Thus, if prevention gaps among a given key population are small (e.g. high relative HIV treatment initiation rates and high levels of condom use), then the tPAF_10_ from 2019 to 2028 may be small assuming intervention coverage is maintained at levels achieved by 2019. As an example, Figure 1C depicts the tPAF_10_ of prevention among FSW if conditions are maintained, whereas Figure 1D depicts the tPAF_10_ if condom‐use among FSW is reduced from 2019 onwards in Yaoundé, Cameroon [13].

Finally, to further interpret heterogeneity in onward transmission risks explicitly as disproportionate risks, the per‐capita tPAF_t_ can be defined by standardizing by the population size of subgroups. Per‐capita tPAF_t_ may be particularly helpful where a small subset of the population may experience disproportionate acquisition and secondary transmission risks in a dense sexual and/or injecting network, but experience limited onward transmission outside the dense network. Thus, the per‐capita tPAF_t_ represents disproportionate onward transmission risks from prevention gaps. For example, over a ten‐year time‐horizon, the tPAF_t_ per 1000 person‐years of prevention gaps among FSW living with HIV was five to ten fold higher than the tPAF_t_ per 1000 person‐years of other women living with HIV and who were not engaged in sex work in studies from Cameroon and Southern Africa [11, 13]. As way of interpretation, this means that unmet needs among FSW may lead to five to ten fold more transmissions per person living with HIV over a ten‐year time‐horizon than those of women not engaged in sex work, such that it may be most efficient to address the unmet needs of FSW. Doing so in practice, however, often calls for tailored strategies that are effective in addressing the prevention gaps in specific subgroups.

### 2.5 Programmatic implications

The tPAF_t_ requires a counterfactual, but it does not specifically simulate the impact of a particular intervention. The corollary of the tPAF_t_ when a specific intervention is examined, is the prevented fraction (the fraction of infections averted over time due to a specific intervention) [12, 24]. However, the tPAF_t_ could help provide insights into the consequence of ongoing vulnerabilities and prevention gaps in the current trajectory of the epidemic. The tPAF_t_ is the maximum population‐level transmission benefit that can be achieved by completely addressing the specific vulnerability and/or prevention gap. For example, the epidemic consequence of ongoing vulnerabilities in the context of sex work signals the maximum impact that could be achieved from partnership‐specific interventions that reduce transmissions in the context of sex work. Meanwhile, the tPAF_t_ of acquisition risk signals interventions such as pre‐exposure prophylaxis and the tPAF_t_ of the prevention gaps via all partnerships among FSW signals combination interventions to reduce both acquisition and transmission risks. The main added value of the distribution of onward transmissions over time is that they avoid underestimating the importance of key populations to a local epidemic and thus, avoid potentially misallocating resources away from key population services. In addition to the traditional metrics of distribution of annual acquisition or annual transmission, the tPAF_t_ provides a medium to longer term perspective for programmes to help guide the strategic design of services and interventions for key populations in particular.

## 3 CONCLUSIONS

The goal of HIV epidemic appraisals has been to better understand local transmission dynamics. Models of various levels of complexity have been used to estimate the contribution of unmet needs across subgroups, including key populations, via the tPAF_t_ [7, 8, 9, 10, 11, 12, 13, 14]. Model complexity in large part will continue to be driven by data on heterogeneities in exposure/network risk across subsets of the population with critical data inputs. However, community‐led efforts including systematic collection of survey and programmatic data among key populations have been increasing over the last ten years [36, 37, 38]. Another potential benefit of using metrics such as the tPAF_t_ is that by focusing on prevention gaps in the causal pathway, HIV epidemic appraisals may further shift the narrative away from the “contribution” of a subgroup to an epidemic, and assumptions of homogeneity with constructs of a “general” population [19]. Models estimating the tPAF_t_ offer an opportunity to support data‐driven and programme‐relevant appraisals of disproportionate onward transmission risks, alongside traditional metrics of heterogeneity such as annual distribution of acquisitions or transmissions or relative incidence and prevalence, which together can serve as a platform to increase the specificity of a local HIV response. Such metrics could and are being used alongside intervention modelling and economic evaluations. Interpreting and using appraisals of onward and disproportionate transmission risks to inform local HIV programmes includes careful consideration of the past, current and future prevention gaps upon which tPAF_t_ is based.

## Competing interests

The authors have declared no conflict of interest..

## Authors’ contributions

SM, RS, JK, LW, MCB and SB conceptualized the study and wrote the manuscript. RS and JK developed the figures and adaptation from published models with MCB, JK and SB. All contributed to study design, data analyses, interpretation of findings and manuscript editing.
